# Effect of Remimazolam- versus Propofol-Based Total Intravenous General Anesthesia on Intraoperative Hemodynamic Stability for Major Spine Surgery in the Prone Position: A Randomized Controlled Trial

**DOI:** 10.3390/medicina60030432

**Published:** 2024-03-05

**Authors:** Ha-Jung Kim, Ji-Young Kim, Hyeok-Seong Park, Hyungtae Kim, Young-Jin Ro, Won Uk Koh

**Affiliations:** 1 Department of Anesthesiology and Pain Medicine, Asan Medical Center, University of Ulsan College of Medicine, Seoul 05505, Republic of Korea; d100262@amc.seoul.kr (H.-J.K.); kimg02w@amc.seoul.kr (J.-Y.K.); usgra@amc.seoul.kr (H.K.); yjro@amc.seoul.kr (Y.-J.R.); 2 Department of Anesthesiology and Pain Medicine, Shihwa Medical Center, Siheung 15034, Republic of Korea; phsung509@gmail.com

**Keywords:** perioperative hypotension, prone position, propofol, remimazolam, spinal surgery

## Abstract

*Background and Objectives*: Remimazolam offers advantages over propofol in terms of hemodynamic stability. However, it remains unclear whether remimazolam-based total intravenous anesthesia (TIVA) can reduce intraoperative hypotension compared to propofol-based TIVA, especially after prone positioning. In this study, we compared the effects of remimazolam- and propofol-based TIVA on intraoperative hemodynamic stability in patients undergoing surgery in the prone position. *Materials and Methods*: This study randomly assigned patients undergoing major spinal surgery in the prone position to the propofol or remimazolam group. Target-controlled infusion (2–3.5 μg/mL for induction and 2–3 μg/mL for maintenance) was used in the propofol group and continuous infusion (6 mg/kg/h for induction and 1–2 mg/kg/h for maintenance) was used in the remimazolam group; target-controlled infusion (3–5 ng/mL) of remifentanil was performed in both groups. The primary outcomes were the incidence of hypotensive episodes during the first hour after prone positioning. The secondary outcomes included the incidence of severe hypotension and the total amount of inotropic or vasopressor medication. Systolic and mean arterial pressure, heart rate, cardiac index and output, stroke volume, stroke volume variation, and pleth variability index were also evaluated. These variables were recorded per minute for the first 10 min after prone positioning, and every 10 min thereafter. *Results*: The study enrolled 94 patients (47 patients in each group). The incidence of hypotension or severe hypotension did not differ significantly between the two groups during the first hour after prone positioning. The total amount of ephedrine administered during the first hour after prone positioning was lesser (*p* = 0.020) and the mean arterial pressure during the initial 10 min after prone positioning was higher in the remimazolam group (*p* = 0.003). *Conclusions*: Our study uncovered no significant differences in the incidence of hypotension between remimazolam- and propofol-based TIVA in patients undergoing major spine surgery in prone position.

## 1. Introduction

Perioperative hypotension is associated with cardiovascular events, acute kidney injury, and higher one-year mortality [1,2,3]. Several studies have shown that even brief episodes of intraoperative hypotension can have ramifications, highlighting the importance of timely treatment during general anesthesia [4].

Spinal surgery is commonly performed in the prone position, which decreases stroke volume and cardiac index, increasing the risk of hypotension [5]. Neurophysiologic monitoring is often required during spinal surgery, making total intravenous anesthesia (TIVA) more favorable than inhalation agents, due to the minimal effects of the former on the latency or amplitude of somatosensory potentials and motor-evoked potentials observed during intraoperative patient monitoring [6]. However, propofol, the most frequently employed anesthetic agent for TIVA, increases the risk of intraoperative hypotension and often requires inotropic support [7,8].

Remimazolam is a novel, ultrashort-acting benzodiazepine used to induce general anesthesia, which is rapidly hydrolyzed by carboxylesterase-1 into an inactive metabolite [9], making it an appropriate agent for continuous infusion during general anesthesia [10]. There have been case reports suggesting that remimazolam is suitable as an anesthetic agent for spine surgery, as remimazolam shows minimal effect on intraoperative neurophysiologic monitoring [11]. In addition, some previous studies showed that remimazolam-based TIVA had an advantage over propofol-based TIVA in hemodynamic stability [12,13]. Even in hypertensive or elderly patients, remimazolam has shown favorable outcomes in terms of hypotension during both the induction and post-induction periods [14,15]. However, no study has investigated the hemodynamic stability of remimazolam when administered as a general anesthetic in patients undergoing surgery in the prone position. In this study, we hypothesized that remimazolam-based TIVA conferred better hemodynamic stability compared to propofol-based TIVA in the prone position. Therefore, we aimed to investigate the effect of remimazolam versus propofol on the incidence of intraoperative hypotension in patients undergoing major spinal surgery in the prone position.

## 2. Materials and Methods

This single-center, prospective, randomized control trial was performed at Asan Medical Center, a tertiary referral center located in Seoul, South Korea. The study was conducted in accordance with the principles enshrined in the Declaration of Helsinki. The trial was registered at the ClinicalTrials.gov (NCT05644483) website. The study protocol was approved by the Institutional Review Board of Asan Medical Center (#2021-1514; Approval date: 22/OCT/2021). All participants provided written informed consent before enrollment.

### 2.1. Study Population

All patients undergoing major spinal surgery in the prone position at the orthopedic department of our center between March 2022 and January 2023 were considered eligible for the study and screened. Patients aged between 19 and 80 years, with an American Society of Anesthesiologists physical status of 1–3, were included in the study. Patients were excluded if they had uncontrolled hypertension, hypothyroidism, moderate to severe cardiovascular or liver disease, acute narrow-angle glaucoma, shock, acute alcoholism, or body mass index below 15 kg/m^2^ or over 35 kg/m^2^ (Supplementary Table S1). All enrolled patients were randomly allocated to either the propofol group or the remimazolam group in a ratio of 1:1, without being informed of the allocation. Randomization was performed using a computer-generated randomization program (https://sealedenvelope.com, accessed on 1 January 2024) by one researcher who was not involved in the anesthetic management of the study patients. Group assignments were obscured in sealed opaque envelopes and opened immediately before anesthetic induction, and subsequently, nurses were informed of these assignments to prepare the anesthetic agents. Due to the color difference of the two study drugs, blinding the investigators was impossible. However, the type of study anesthetic infused (propofol or remimazolam) was concealed from the patients.

### 2.2. Anesthesia

Upon arrival at the operating room, the patients underwent standard monitoring, including pulse oximetry, pleth variability index (PVi), non-invasive blood pressure (NIBP) measurement, and electrocardiography. NIBP measurement was performed in one arm at 3 min intervals before arterial cannula insertion. Pulse oximetry and PVi were monitored continuously using Rainbow sensor (Masimo Corp., Irvine, CA, USA) at the contralateral arm; continuous electrocardiography monitoring was also performed. Induction was achieved using remimazolam (ByFavo, Hana Pharmaceutical, Seoul, Republic of Korea) at a rate of 6 mg/kg/h in the remimazolam group or 2% propofol (Fresofol^®^, Fresenius Kabi Austria GmbH, Graz, Austria). target-controlled infusion using the Schneider pharmacokinetic model at an effect-site concentration of 2.0 μg/mL in the propofol group. The propofol infusion rate was increased by 0.5 μg/mL every 30 s until loss of consciousness occurred. Remifentanil was infused at a rate of 3–5 ng/mL using target-controlled infusion following Minto algorithm in both groups. After loss of consciousness, the infusion rate was reduced to 1–2 mg/kg/h for remimazolam or 2–3 μg/mL for propofol target-controlled infusion. Mask ventilation was delivered using rocuronium at a dose of 0.6–0.8 mg/kg in both groups and tracheal intubation was performed after adequate relaxation. During induction, a crystalloid solution was administered at a rate of 6 mL/kg. A 20 g arterial cannula was inserted into the radial artery and an 18 g intravenous cannula was inserted after anesthetic induction. Thereafter, systolic arterial pressure (SAP), mean arterial pressure (MAP), and other hemodynamic variables including cardiac index and output, stroke volume, and stroke volume variation (SVV) were examined continuously. The hemodynamic variables excluding arterial pressure were measured by Rainbow sensor (Masimo Corp., Irvine, CA, USA).

**Figure 1:**
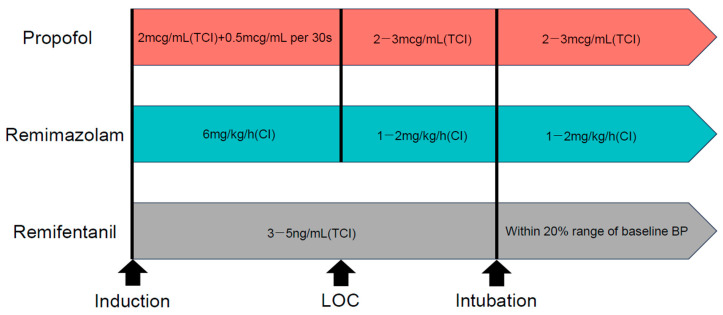
Research protocols. Abbreviation: CI, continuous infusion; TCI, target-controlled infusion; LOC, loss of consciousness; SBP, systolic blood pressure.

After induction of anesthesia, a urinary catheter was inserted, followed by positioning the patient in the prone position on the operating table (ProAxis^®^, Mizuho OSI, Union City, CA, USA). The patients’ upper chest and bilateral iliac crest were supported by the table, allowing the abdomen to hang freely to reduce intra-abdominal pressure. During maintenance, the infusion rates of propofol (2–3 μg/mL) and remimazolam (1–2 mg/kg/h) were adjusted to maintain a patient state index value between 25 and 50, whereas the infusion rate of remifentanil was adjusted to maintain blood pressure (BP) within 20% of baseline (Figure 1).

### 2.3. Definition of Hemodynamic Events and Management

Baseline systolic blood pressure (SBP) was calculated as the average of three ward NIBP measurements at rest. Hypotension was defined as SAP < 80% of the baseline SBP or <90 mmHg, or MAP < 80% of the baseline MBP or <65 mmHg. Severe hypotension was defined as SAP < 70% of the baseline SBP or <80 mmHg, or MAP < 70% of the baseline MBP or <55 mmHg. Post-positioning BP was defined as the arterial BP measured within 10 min of prone positioning. Hypotension was treated by lowering the infusion rate of remifentanil, whereas severe hypotension was treated by the administration of phenylephrine (50–100 mcg) or ephedrine (5–10 mg), irrespective of the trial drug. Continuous infusion of norepinephrine or phenylephrine was initiated if severe hypotensive episodes occurred three times within 15 min or five times within 30 min. The infusion rate of remifentanil was increased if the SAP was elevated to >20% of the baseline SBP. If the SAP increased by >30% from baseline, nicardipine (300 mcg) administration was considered. Bradycardia (heart rate < 40 beats/min) was treated with atropine (0.5 mg), and esmolol (0.5 mg/kg) was administered for tachycardia (heart rate > 100 beats/min).

### 2.4. Outcome Measures

The primary outcomes of this study were the incidence of hypotension during the first hour after prone positioning. As a secondary outcome, we also compared the incidence of severe hypotension, the number of hypotensive and severe hypotensive events per individual during the same time period. The total amount of inotropic or vasopressor medication used to maintain hemodynamic stability for one hour after positioning was designated as a secondary outcome. Additionally, we analyzed continuous hemodynamic variables including SAP and MAP, heart rate, cardiac index and output, stroke volume, SVV, and PVi. All variables were recorded per minute for the first 10 min after prone positioning, and every 10 min thereafter, up to one hour.

### 2.5. Statistical Analysis

In a retrospective review of the medical records of 25 patients who underwent major spine surgery under propofol-based TIVA in prone position at our institution, it was found that hypotension during the first hour after prone positioning occurred in 14 patients (56%). Assuming that administering remimazolam instead of propofol could reduce this incidence by 28%, power analysis indicated that a minimum of 44 patients per group would be required to detect a difference in the incidence of hypotension with a power of 0.8 and an alpha error of 0.1. The final sample size was determined to be 94, accounting for dropouts from each group.

All statistical and graphical analyses were performed using R (version 4.1.2; http://www.rproject.org) and SAS^®^, version 9.4 (SAS Institute Inc., Cary, NC, USA). The significance of the outcome was denoted as two-tailed *p*-values < 0.05. The primary outcome, the incidence of hypotension, was assessed using Student’s *t*-test. Other continuous variables were evaluated using Student’s *t*-test or Wilcoxon’s rank-sum test, as appropriate. Either the chi-square test or Fisher’s exact test was used to analyze categorical data. In addition, linear mixed models were generated to evaluate the longitudinal changes in MAP and heart rate. Using these models, we tested the group and time effects and the interactions of group and time.

## 3. Results

**Figure 2:**
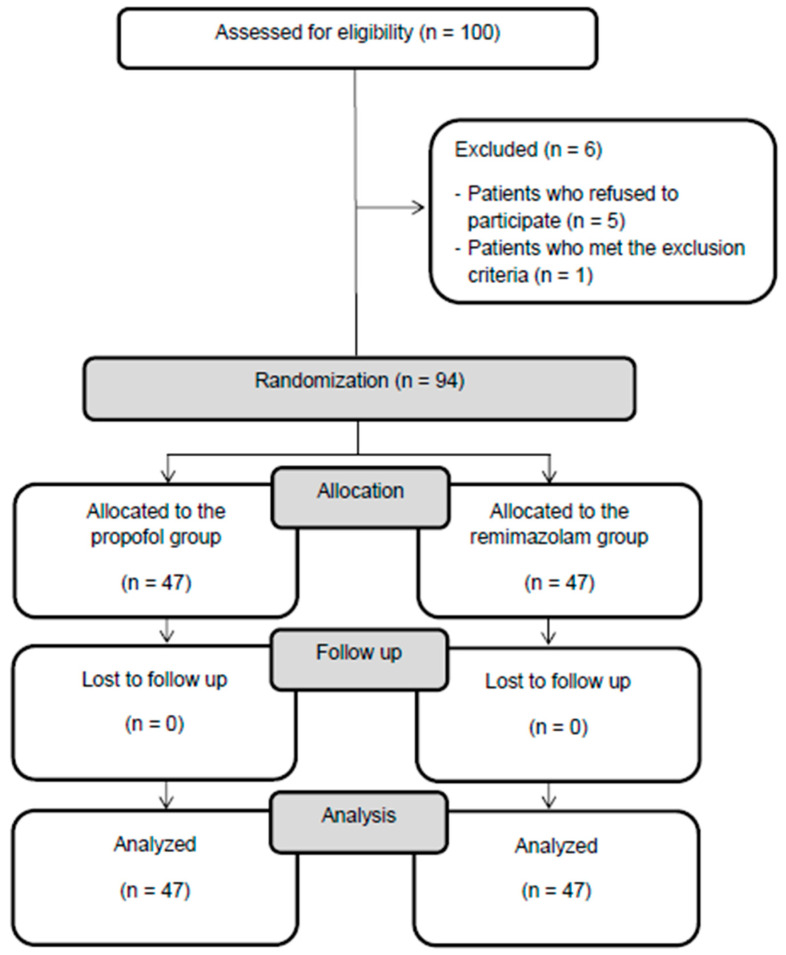
Flow chart of patient enrollment.

We screened 100 patients who were scheduled to undergo major spine surgery in the prone position at our hospital for eligibility. In total, 5 of these patients refused to participate in this study and were excluded, along with 1 patient with uncontrolled hypertension. The remaining 94 patients were enrolled and included in the final analysis, without any dropout (Figure 2). The demographic data, comorbidities, preoperative laboratory data, and anesthesia and surgery-related data of the propofol and remimazolam groups are presented in Table 1. These baseline characteristics did not differ significantly between the two groups, with the exception of weight being higher in the propofol group and a higher prevalence of diabetes mellitus patients in the remimazolam group.

The incidence of hypotension during the first hour after prone positioning, the primary outcome of this study, did not differ between the two groups. Moreover, there were no differences in the incidence of severe hypotension, or the number of hypotensive and severe hypotensive events per individual during the first hour of prone positioning. The total amount of ephedrine administered during the first hour after prone positioning was greater in the propofol group than that in the remimazolam group (*p* = 0.020). No significant differences were observed with any of the other drugs (Table 2).

Continuous variables were compared using Wilcoxon’s rank sum test, and the categorical variables were analyzed using Chi-square test or Fisher’s exact test.

**Figure 3:**
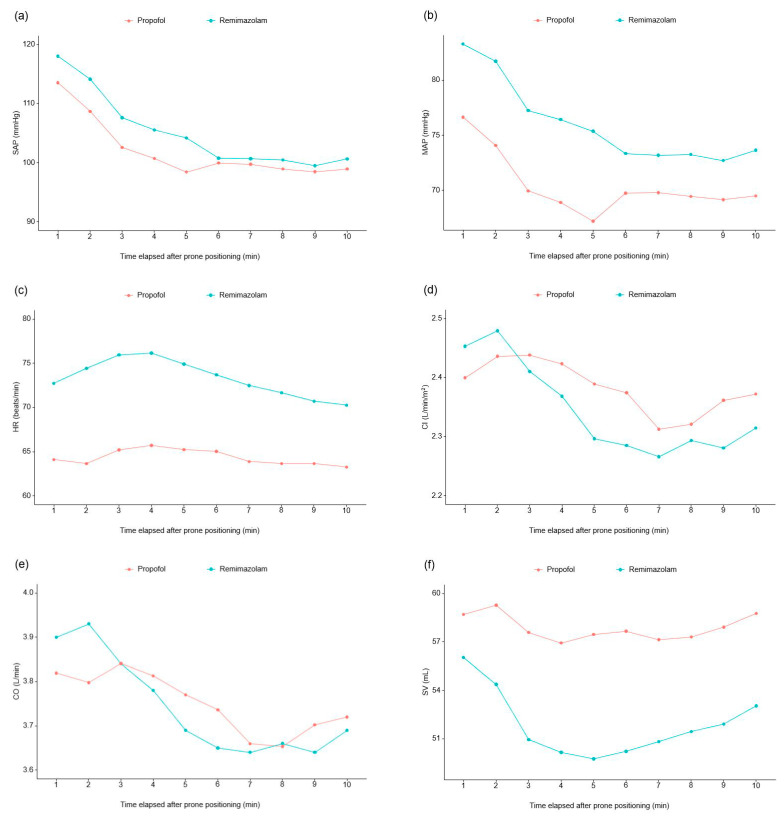

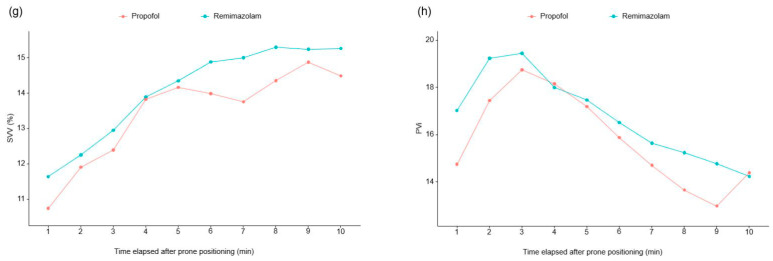
Serial changes in systolic arterial pressure (**a**), mean arterial pressure (**b**), heart rate (**c**), cardiac index (**d**), cardiac output (**e**), stroke volume (**f**), stroke volume variant (**g**) and pleth variability index (**h**) during the initial 10 min after prone positioning. Abbreviations: SAP, systolic arterial pressure; MAP, mean arterial pressure; HR, heart rate; CI, cardiac index; CO, cardiac output; SV, stroke volume; SVV, stroke volume variant; PVi, pleth variability index.

In the initial 10 min after prone positioning (Supplementary Table S2), the analysis of hemodynamic variables using linear mixed model demonstrated that the interaction of group and time had a significant effect on heart rate (*p* = 0.029). The group effect was significant in terms of MAP, heart rate, and stroke volume; the remimazolam group showed a significantly higher MAP (*p* = 0.003) and heart rate (*p* < 0.001) compared to the propofol group, while stroke volume was significantly higher in the propofol group compared with the remimazolam group (*p* = 0.029) during the first 10 min following prone positioning (Figure 3). The other secondary hemodynamic parameters including cardiac output and index, SVV, and PVi during the initial 10 min after prone positioning did not differ between the study groups.

**Figure 4:**
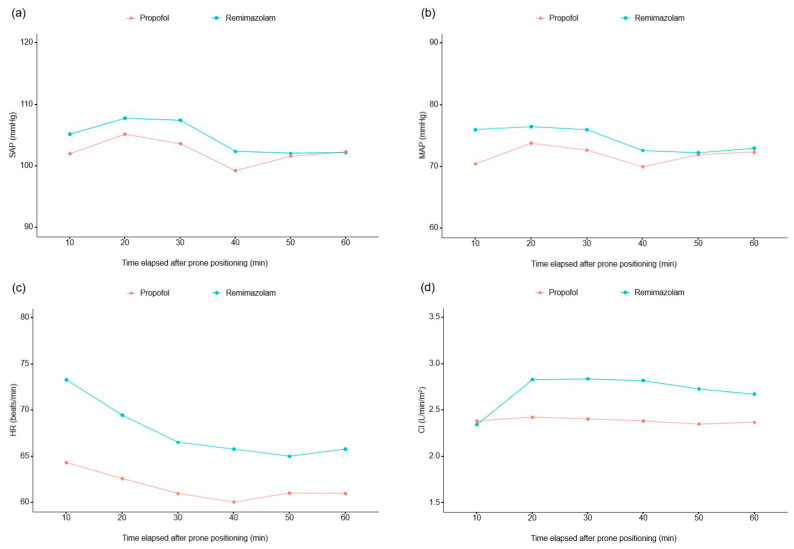

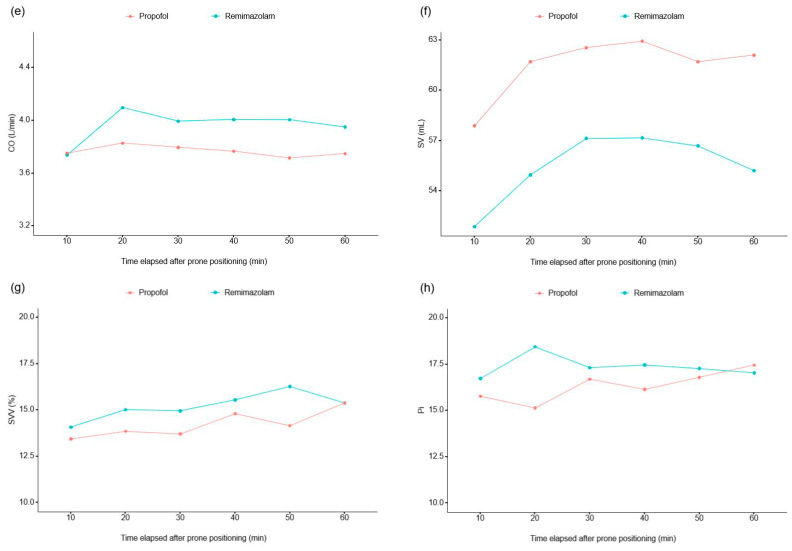
Serial changes in (**a**) systolic arterial pressure, (**b**) mean arterial pressure, (**c**) heart rate, (**d**) cardiac index, (**e**) cardiac output, (**f**) stroke volume, (**g**) stroke volume variation and (**h**) pleth variability index during the first hour after prone positioning. Abbreviations: SAP, systolic arterial pressure; MAP, mean arterial pressure; HR, heart rate; CI, cardiac index; CO, cardiac output; SV, stroke volume; SVV, stroke volume variation; PVi, pleth variability index.

In the analysis of one hour of hemodynamic data measured at 10 min intervals from the first 10 min following the change to prone position (Supplementary Table S3), the interaction of group and time had no significant effect on any hemodynamic data except for heart rate (*p* = 0.030). The remimazolam group exhibited a significantly greater heart rate (*p* = 0.003) and a significantly lower stroke volume (*p* = 0.046) compared to the propofol group; no significant differences were observed in the other secondary hemodynamic parameters (Figure 4).

## 4. Discussion

In this randomized controlled study, we compared the effects of remimazolam- and propofol-based TIVA on intraoperative hemodynamic stability during major spine surgery in the prone position. Our study revealed no significant difference in the incidence of hypotension between the two groups. However, the MAP during the initial 10 min after prone positioning was higher in the remimazolam group.

Previous studies have reported a lower incidence of intraoperative hemodynamic instability with remimazolam compared to propofol [12,13,16,17]. This result may be attributed to the fact that remimazolam has no effect, whereas propofol has a relatively large suppressive effect on the cardiovascular system. However, they compared the two drugs in supine or lateral decubitus positioned patients, which is the major difference between the previous and current studies. The prone position itself has a significant impact on physiology, particularly on the respiratory and cardiovascular systems [5]. When a patient is made prone during anesthesia, the cardiac output may decline due to the reduction in stroke volume. The reduction in stroke volume is thought to be due to the reduction in pre-load, which can be caused by blood sequestration, caval compression, amplified intra-thoracic pressure, use of positive pressure ventilation, and positive end-expiratory pressure [5]. This decline in stroke volume leads to a decrease in arterial pressure, which is partially countered by escalation in the heart rate and peripheral vascular resistance. Based on our research findings, we speculate that the effect of the prone position itself on hemodynamic stability may outweigh the effect of anesthetic agents in patients undergoing surgery in the prone position.

However, there was a difference in the total amount of ephedrine administered during the first hour after assuming the prone position between the two groups. This can be attributed to the dominant parasympathetic effect of propofol and the dominant sympathetic effect of midazolam during sedation [18,19]. The propofol group exhibited a lower heart rate due to the drug’s parasympathetic effect, resulting in a lower heart rate during hypotensive events and more frequent administration of ephedrine. Therefore, the difference in the ephedrine dose suggests that remimazolam provides hemodynamic stability during TIVA in prone-positioned patients.

As a secondary outcome measure, we compared the continuously monitored hemodynamic variables such as SAP and MAP, heart rate, cardiac index, cardiac output, stroke volume, SVV and PVi between the two groups. Our results indicated that the propofol group showed a significantly lower MAP compared to the remimazolam group, along with a lower heart rate and elevated stroke volume during the immediate post-positioning period. This result could be linked to the compensatory venous relaxation in the propofol group [20]. As cardiac output is the product of stroke volume and heart rate, changes in these two factors may have resulted in no significant difference in the cardiac output between the two groups. However, our study did not compare the effects of remimazolam and propofol on systemic vascular resistance changes in the prone position. Previous studies have suggested that remimazolam can maintain relatively higher SVR levels in patients in the supine position [15]. Therefore, further research is needed to investigate the impact of drugs on SVR in patients in the prone position.

On the other hand, in another study comparing remimazolam and propofol, it was reported that there were no differences in hemodynamic indices, including MAP [21]. The authors speculated that the inconsistency with other studies may be attributed to differences in the administered drug regimen [21]. Similarly, in our study, the possibility of disparities arising from the drug regimen cannot be excluded. Particularly, during the design phase of our study, there was limited research available on remimazolam, leading us to set the dosage at 1–2 mg/kg/h. However, subsequent studies published later demonstrated that effective general anesthesia could be maintained even at lower dosages [22]. Furthermore, in this study, the dosage of remifentanil was adjusted based on hemodynamic changes rather than nociception indicators including surgical pleth index. However, since there is controversy regarding the benefits of surgical pleth index [23,24], and the control of the dosage was adjusted according to the same criteria, it is unlikely to have had a significant impact on our results. Nevertheless, considering the potential impact of differences in drug regimens on hemodynamic indices in the prone position, further research is required.

The primary objective of perioperative BP management is to ensure adequate organ perfusion. Organ perfusion pressure is determined by the difference between the inflow and outflow pressures, with MAP serving as the inflow pressure for most organs and acting as a clinically available surrogate for perfusion pressure [25]. Although there is an ongoing debate about whether hypotension should be defined based on absolute or relative BP [25,26,27,28], there is consensus that MAP below absolute thresholds is progressively associated with myocardial and renal injury, with prolonged exposure at any given threshold increasing the risk of hypoperfusion injury [3,28]. It is important to note that transient episodes of hypotension, where the MAP falls below thresholds of 50–65 mmHg, are associated with renal and myocardial injury [28]. Maheshwari et al. stated that during elective non-cardiac surgery, one-third of hypotensive episodes occurred before incision and were significantly associated with acute kidney injury [29]. In our study, we observed that the group receiving propofol had a lower MAP as compared to the group receiving remimazolam in the immediate post-positioning period. These findings are noteworthy because maintaining MAP above a certain threshold is important to prevent adverse outcomes such as acute kidney injury and myocardial injury. Given that hypotension between anesthetic induction and surgical incision is preventable and is associated with ischemic injuries, it is imperative to consider it a modifiable risk factor and implement measures for its prevention. Nevertheless, our results suggest that remimazolam may be a better option over propofol for preserving MAP during spinal surgery in the period immediately after prone positioning, which is particularly significant, because even brief episodes of hypotension have been associated with renal and myocardial injury.

Our study has some limitations. We did not investigate whether the difference in MAP between the two groups affected the long-term major postoperative complications. Additionally, we did not establish a specific cutoff value for meaningful hypotensive episodes and BP thresholds. As we applied our study protocol to only up to one hour after changing to the prone position, we did not compare postoperative complications. There would be numerous confounding factors when comparing the incidence of postoperative complications between the two groups, as various factors beyond this protocol period such as bleeding or surgery and anesthetic time could have influenced postoperative complications. However, we limited our protocol to only up to one hour after turning the patient prone considering the potential association between bleeding occurring during the main surgical procedure and the occurrence of hypotension. Further research is needed to investigate whether the type of anesthetic agent influenced the incidence of hypotension and postoperative complications across a more homogenous group throughout the entire duration. Second, the generalizability of our findings may be limited due to the single-center design and relatively small sample size. In addition, we excluded the patients with extreme body mass index or those with high-risk of ASA PS 4 or higher. Obese patients were particularly at high risk of hemodynamic instability associated with the increased abdominal pressure and decreased venous return [30]. As the prevalence of obese and high-risk patients increases, further research into whether anesthetic agents impact the incidence of hypotension in patients, including these groups, in the prone position. Third, despite random allocation of patients, differences were observed in terms of weight and the number of patients with diabetes mellitus. However, the differences in weight were not clinically significant, and it is unlikely that the presence of diabetes had a decisive impact on the results of our study [31].

## 5. Conclusions

In conclusion, our study uncovered no significant differences in the incidence of hypotension during the first hour after prone positioning between remimazolam- and propofol-based TIVA. This may be attributed to the likelihood that the influence of the prone position itself on hemodynamic indices is greater than that of the anesthetic drugs. However, given the potential adverse effects of transient hypotension on patient outcomes, prioritizing remimazolam, which helps maintain a higher MAP during the initial 10 min following prone positioning, could be a viable consideration.

## Figures and Tables

**Table 1:** Patient baseline characteristics.

	Propofol Group (n = 47)	Remimazolam Group (n = 47)	*p*-Value
Demographic data			
Sex (male)	20 (42.6%)	15 (31.9%)	0.286
ASA PS			
2	42 (89.4%)	43 (91.5%)	
3	5 (10.6%)	4 (8.5%)	
Age (years)	67.2 ± 7.5	67.4 ± 8.0	0.873
Height (m)	1.6 ± 0.1	1.6 ± 0.1	0.927
Weight (kg)	64.9 ± 9.2	61.1 ± 9.0	0.048
Medical history			
Diabetes mellitus	7 (14.9%)	16 (34.0%)	0.031
Hypertension	27 (57.4%)	23 (48.9%)	0.408
Ischemic heart disease	4 (8.5%)	2 (4.3%)	0.677
Cerebrovascular accident	2 (4.3%)	0 (0.0%)	0.495
Pulmonary disease	4 (8.5%)	4 (8.5%)	1.000
Renal disease	1 (2.1%)	4 (8.5%)	0.361
Preoperative laboratory data			
Hemoglobin (g/dL)	13.0 ± 1.4	12.7 ± 1.6	0.374
White blood cell (10^3^/µL)	6.4 ± 1.5	6.3 ± 2.1	0.681
Platelet (10^9^/µL)	235.6 ± 49.8	235.8 ± 53.8	0.986
Albumin (g/dL)	3.8 ± 0.3	4.5 ± 4.7	0.803
Creatinine (mg/dL)	0.8 ± 0.2	0.8 ± 0.2	0.714
Aspartate transaminase (IU/L)	23.1 ± 7.1	23.1 ± 6.3	0.976
Alanine transaminase (IU/L)	21.8 ± 11.5	20.3 ± 8.0	0.462
C-reactive protein (mg/dL)	0.2 ± 0.6	0.3 ± 0.8	0.506
Anesthesia and surgery-related data			
Anesthetic time (min) *	−189 (170–210)	186 (170–210)	0.623
Operative time (min) *	137 (117–157)	135 (115–159)	0.877
Total amount of remifentanil (mcg) *	1600 (1300–1987)	1562.5 (1226–2150)	0.715

All data are expressed as the mean ± standard deviation, median (interquartile range), or n (%). Abbreviation: ASA PS, American Society of Anesthesiologists Physical Status; *, Wilcoxon’s rank sum test was used for the analysis of the variables. Other continuous variables were compared using Student’s *t*-test, and the categorical variables were analyzed using Chi-square test or Fisher’s exact test.

**Table 2:** Intraoperative hemodynamic events and vasopressor requirement during the first hour after prone positioning.

	Propofol Group (n = 47)	Remimazolam Group (n = 47)	*p*-Value
Intraoperative adverse events			
Patients with hypotensive event(s)	45 (95.7%)	39 (83.0%)	0.091
Hypotensive event per individual	4.7 (2.4%)	4.1 (2.7%)	0.366
Patients with severe hypotensive event(s)	36 (76.6%)	31 (66.0%)	0.254
Severe hypotensive event(s) per individual	2.8 (2.3%)	2.1 (2.1%)	0.128
Amount of administered inotropics or vasopressors			
Total amount of ephedrine (mg)	5 (0–10)	0 (0–5)	0.020
Total amount of phenylephrine (mcg)	1200 (0–2950)	325 (0–1700)	0.239
Total amount of norepinephrine (mcg)	0 (0–0)	0 (0–0)	0.164

All data are expressed as the median (interquartile range), or n (%).

## Data Availability

The data that support the findings of this study are available from the corresponding author upon reasonable request.

## Supplementary Materials

The following supporting information can be downloaded at: https://www.mdpi.com/article/10.3390/medicina60030432/s1. Supplementary Table S1. Inclusion and exclusion criteria of this study; Supplementary Table S2a–h. Longitudinal hemodynamic data analysis using linear mixed model during the first 10 min after prone positioning. Supplementary Table S3a–h. Longitudinal hemodynamic data analysis using linear mixed model during the first hour after prone positioning.
