# Supplementary material for: Effect of Remimazolam- versus Propofol-Based Total Intravenous General Anesthesia on Intraoperative Hemodynamic Stability for Major Spine Surgery in the Prone Position: A Randomized Controlled Trial
Source: Medicina (Kaunas). 2024 Mar 5;60(3):432. doi: 10.3390/medicina60030432 (PMC10972152; doi:10.3390/medicina60030432)
Supplement: Supplementary file 1 [file medicina-60-00432-s001.zip › medicina-2875946-supplementary.pdf]

Supplementary Table S1. Participation inclusion and exclusion criteria

Inclusion criteria	Exclusion criteria
Aged between 19 and 80 years	Uncontrolled hypertension
American Society of Anesthesiologists physical status 1-3	Hypothyroidism
	Moderate to severe cardiovascular or liver disease
	Acute narrow-angle glaucoma
	Shock
	Acute alcoholism
	Body mass index below $15kg/m^2$ or over $35kg/m^2$

Supplementary Table S2a-h. Longitudinal hemodynamic data analysis using linear mixed model during the first 10 min after prone positioning.

a. Systolic arterial pressure (mmHg)

Time point	Propofol group (n=47)		Remimazolam group (n=47)		p-value
	LSmean	SE	LSmean	SE	
1 min	113.53	3.32	118.04	3.32	0.384*
2 min	108.70	3.41	114.15	3.41	<0.001†
3 min	102.60	3.36	107.62	3.36	0.260‡
4 min	100.74	2.98	105.55	2.98	
5 min	98.43	2.95	104.19	2.95	
6 min	99.96	2.75	100.77	2.75	
7 min	99.74	2.76	100.68	2.76	
8 min	98.94	2.90	100.45	2.90	
9 min	98.49	2.84	99.49	2.84	
10 min	98.94	2.86	100.66	2.86	

b. Mean arterial pressure (mmHg)

Time point	Propofol group (n=47)		Remimazolam group (n=47)		p-value
	LSmean	SE	LSmean	SE	
1 min	76.64	2.18	83.28	2.18	0.004*
2 min	74.06	2.14	81.72	2.14	<0.001†
3 min	69.96	1.90	77.26	1.90	0.109‡
4 min	68.89	1.72	76.43	1.72	
5 min	67.19	1.71	75.36	1.71	
6 min	69.74	1.57	73.34	1.57	
7 min	69.79	1.58	73.19	1.58	
8 min	69.45	1.68	73.26	1.68	
9 min	69.15	1.64	72.70	1.64	
10 min	69.49	1.61	73.64	1.61	

c. Heart rate (beats/min)

Time point	Propofol group (n=47)	Remimazolam group (n=47)	p-value

	LSmean	SE	LSmean	SE	
1 min	64.09	1.92	72.74	1.92	<0.001*
2 min	63.64	1.83	74.43	1.83	<0.001†
3 min	65.19	1.67	75.94	1.67	0.029‡
4 min	65.70	1.64	76.15	1.64	
5 min	65.23	1.62	74.91	1.62	
6 min	65.02	1.62	73.68	1.62	
7 min	63.89	1.54	72.49	1.54	
8 min	63.64	1.54	71.66	1.54	
9 min	63.64	1.57	70.70	1.57	
10 min	63.26	1.59	70.26	1.59	

d. Cardiac index (L/min/m²)

Time point	Propofol group (n=47)		Remimazolam group (n=47)		p-value
	LSmean	SE	LSmean	SE	
1 min	2.40	0.08	2.45	0.08	0.712*
2 min	2.44	0.09	2.48	0.09	0.004†
3 min	2.44	0.08	2.41	0.08	0.258‡
4 min	2.42	0.08	2.37	0.08	
5 min	2.39	0.08	2.30	0.08	
6 min	2.37	0.07	2.29	0.07	
7 min	2.31	0.07	2.27	0.07	
8 min	2.32	0.07	2.29	0.07	
9 min	2.36	0.08	2.28	0.08	
10 min	2.37	0.07	2.31	0.07	

e. Cardiac output (L/min)

Time point	Propofol group (n=47)		Remimazolam group (n=47)		p-value
	LSmean	SE	LSmean	SE	
1 min	3.82	0.13	3.90	0.13	0.949*
2 min	3.80	0.13	3.93	0.13	0.023†
3 min	3.84	0.13	3.84	0.13	0.339‡
4 min	3.81	0.12	3.78	0.12	

5 min	3.77	0.12	3.69	0.12	
6 min	3.74	0.11	3.65	0.11	
7 min	3.66	0.11	3.64	0.11	
8 min	3.65	0.12	3.66	0.12	
9 min	3.70	0.12	3.64	0.12	
10 min	3.72	0.11	3.69	0.11	

f. Stroke volume (mL)

Time point	Propofol group (n=47)		Remimazolam group (n=47)		p-value
	LSmean	SE	LSmean	SE	
1 min	58.70	2.22	56.03	2.22	0.029*
2 min	59.28	2.26	54.37	2.27	<0.001†
3 min	57.58	2.12	50.95	2.13	0.195‡
4 min	56.93	1.95	50.15	1.96	
5 min	57.46	2.05	49.75	2.05	
6 min	57.66	1.97	50.22	1.97	
7 min	57.14	1.94	50.82	1.94	
8 min	57.30	1.96	51.45	1.96	
9 min	57.92	1.94	51.91	1.94	
10 min	58.76	1.96	53.04	1.96	

g. Stroke volume variation (%)

Time point	Propofol group (n=47)		Remimazolam group (n=47)		p-value
	LSmean	SE	LSmean	SE	
1 min	10.74	1.03	11.64	1.03	0.656*
2 min	11.90	1.15	12.25	1.16	<0.001†
3 min	12.39	0.98	12.95	0.98	0.523‡
4 min	13.82	1.07	13.89	1.07	
5 min	14.16	1.10	14.35	1.10	
6 min	13.99	1.07	14.88	1.07	
7 min	13.76	1.01	15.00	1.01	
8 min	14.35	1.06	15.30	1.06	
9 min	14.87	1.09	15.24	1.09	

10 min	14.49	1.10	15.26	1.10

h. Pleth variability index

Time point	Propofol group (n=47)		Remimazolam group (n=47)		p-value
	LSmean	SE	LSmean	SE	
1 min	14.74	1.17	17.03	1.19	0.423*
2 min	17.44	1.23	19.23	1.24	<0.001†
3 min	18.74	1.19	19.45	1.19	0.391‡
4 min	18.15	1.12	18.00	1.12	
5 min	17.19	1.12	17.47	1.12	
6 min	15.87	1.04	16.51	1.04	
7 min	14.70	0.94	15.64	0.94	
8 min	13.66	0.86	15.23	0.86	
9 min	12.98	0.83	14.77	0.83	
10 min	14.38	1.55	14.23	1.55	

LSmean, least square mean; SE, standard error.
*, group effect; †, time effect; ‡, interaction of group & time

Supplementary Table S3a-h. Longitudinal hemodynamic data analysis using linear mixed model during the first hour after prone positioning.

a. Systolic arterial pressure (mmHg)

Time point	Propofol group (n=47)		Remimazolam group (n=47)		p-value
	LSmean	SE	LSmean	SE	
10 min	102.01	2.55	105.16	2.55	0.499*
20 min	105.17	2.95	107.76	2.95	0.001†
30 min	103.62	2.94	107.43	2.94	0.708‡
40 min	99.28	2.87	102.39	2.87	
50 min	101.6	2.63	102.07	2.63	
60 min	102.32	2.51	102.18	2.51	

b. Mean arterial pressure (mmHg)

	Propofol group (n=47)		Remimazolam group (n=47)		p-value
	LSmean	SE	LSmean	SE	
10 min	70.44	1.32	76.02	1.32	0.198*
20 min	73.79	1.86	76.46	1.86	0.003†
30 min	72.66	1.95	75.99	1.95	0.276‡
40 min	69.98	1.91	72.63	1.91	
50 min	71.94	1.72	72.28	1.72	
60 min	72.36	1.67	72.99	1.67	

c. Heart rate (beats/min)

Time point	Propofol group (n=47)		Remimazolam group (n=47)		p-value
	LSmean	SE	LSmean	SE	
10 min	64.33	1.54	73.30	1.54	0.003*
20 min	62.62	1.48	69.47	1.48	<0.001†
30 min	61.00	1.47	66.53	1.47	0.030‡
40 min	60.06	1.46	65.79	1.46	
50 min	61.04	1.44	65.02	1.44	
60 min	61.00	1.39	65.79	1.39	

d. Cardiac index (L/min/m²)

Time point	Propofol group (n=47)		Remimazolam group (n=47)		p-value
	LSmean	SE	LSmean	SE	
10 min	2.38	0.07	2.34	0.07	0.440*
20 min	2.42	0.36	2.83	0.36	0.010†
30 min	2.40	0.36	2.84	0.36	0.445‡
40 min	2.38	0.36	2.82	0.36	
50 min	2.35	0.34	2.73	0.34	
60 min	2.37	0.31	2.67	0.31	

e. Cardiac ouput (L/min)

Time point	Propofol group (n=47)		Remimazolam group (n=47)		p-value
	LSmean	SE	LSmean	SE	
10 min	3.75	0.11	3.74	0.11	0.545*
20 min	3.83	0.29	4.10	0.29	0.020†
30 min	3.80	0.21	3.99	0.21	0.594‡
40 min	3.77	0.25	4.01	0.25	
50 min	3.71	0.31	4.00	0.31	
60 min	3.75	0.29	3.95	0.29	

f. Stroke volume (mL)

Time point	Propofol group (n=47)		Remimazolam group (n=47)		p-value
	LSmean	SE	LSmean	SE	
10 min	57.87	1.91	51.87	1.91	0.046*
20 min	61.71	2.23	54.96	2.23	<0.001†
30 min	62.55	2.23	57.13	2.23	0.332‡
40 min	62.93	2.19	57.17	2.19	
50 min	61.71	2.38	56.68	2.38	
60 min	62.10	2.19	55.21	2.19	

g. Stroke volume variation (%)

Time point	Propofol group (n=47)		Remimazolam group (n=47)		p-value
	LSmean	SE	LSmean	SE	
10 min	13.45	0.99	14.07	0.99	0.573*
20 min	13.86	1.26	15.02	1.26	0.002†
30 min	13.71	1.23	14.96	1.23	0.254‡
40 min	14.81	1.25	15.55	1.25	
50 min	14.16	1.52	16.27	1.52	
60 min	15.38	1.41	15.38	1.41	

h. Pleth variability index

Time point	Propofol group (n=47)		Remimazolam group (n=47)		p-value
	LSmean	SE	LSmean	SE	
10 min	15.78	0.85	16.73	0.85	0.495*
20 min	15.15	1.14	18.45	1.14	0.886†
30 min	16.69	1.30	17.32	1.30	0.112‡
40 min	16.14	1.32	17.46	1.32	
50 min	16.79	1.30	17.27	1.29	
60 min	17.45	1.43	17.04	1.42	

LSmean, least square mean; SE, standard error.

*, group effect; †, time effect; ‡, interaction of group & time
